# Influenza Vaccine Effectiveness in Australia During 2017–2019

**DOI:** 10.1111/irv.70137

**Published:** 2025-07-16

**Authors:** Tanya Diefenbach‐Elstob, Monique B. Chilver, Violeta Spirkoska, Kylie S. Carville, Clyde Dapat, Mark Turra, Thomas Tran, Yi‐Mo Deng, Heidi Peck, Ian G. Barr, Nigel Stocks, Sheena G. Sullivan

**Affiliations:** ^1^ WHO Collaborating Centre for Reference and Research on Influenza, Royal Melbourne Hospital at the Peter Doherty Institute for Infection and Immunity Melbourne Victoria Australia; ^2^ Australian Sentinel Practices Research Network (ASPREN), Discipline of General Practice, Faculty of Health and Medical Sciences The University of Adelaide Adelaide South Australia Australia; ^3^ Victorian Influenza Sentinel Practices Network (VicSPIN), Victorian Infectious Diseases Reference Laboratory, Royal Melbourne Hospital at the Peter Doherty Institute for Infection and Immunity Melbourne Victoria Australia; ^4^ Department of Infectious Diseases, University of Melbourne at the Peter Doherty Institute for Infection and Immunity Melbourne Victoria Australia; ^5^ Microbiology and Infectious Diseases, Public Health Laboratory SA Pathology Adelaide South Australia Australia; ^6^ Victorian Infectious Diseases Reference Laboratory, Royal Melbourne Hospital At the Peter Doherty Institute for Infection and Immunity Melbourne Victoria Australia; ^7^ Department of Microbiology and Immunology University of Melbourne, at the Peter Doherty Institute for Infection and Immunity Melbourne Victoria Australia; ^8^ School of Clinical Sciences Monash University Clayton Victoria Australia

**Keywords:** case–control study, influenza, surveillance, test‐negative design, vaccine effectiveness

## Abstract

**Background:**

Vaccine effectiveness (VE) estimates provide important post‐marketing assessment of how well seasonal influenza vaccines prevent medically attended influenza disease. We present VE estimates for primary care in Australia for the 2017–2019 seasons.

**Methods:**

The study used a test‐negative design. Influenza VE was estimated from adjusted logistic regression models comparing the odds of vaccination among influenza‐test‐positive cases and test‐negative non‐cases. Estimates were made overall and separately by influenza type, subtype, lineage and clade and stratified by age group. Antigenic similarity of influenza viruses to vaccine strains was assessed using the haemagglutination inhibition assay, and phylogenetic analysis was performed on sequenced viruses.

**Results:**

The study included 2879, 1973 and 3371 general practice patients with swabs collected during 2017, 2018 and 2019 respectively. Influenza A(H3N2) was predominant in 2017 and 2019, while influenza A(H1N1)pdm09 predominated in 2018. VE was estimated at 37% (95% CI 22, 48) for the 2017 season, 53% (95% CI 33, 67) for 2018 and 50% (95% CI 40, 58) for 2019. In general, estimates were higher against A(H1N1)pdm09 and influenza B viruses and lower against A(H3N2) viruses. Across the three seasons, antigenic data identified a greater proportion of A(H1N1)pdm09 and influenza B viruses than A(H3N2) viruses as antigenically similar to the cell‐propagated reference viruses. VE estimates by clade generally indicated higher VE among viruses in the same clade as the vaccine viruses.

**Conclusions:**

Influenza VE varied across influenza seasons and by influenza type/subtype. Given the ongoing evolution of circulating influenza viruses, vaccine improvements are needed, especially for influenza A(H3N2).

## 1 Background

Circulating influenza viruses constantly evolve due to genetic changes in the surface glycoproteins of the virus to avoid pre‐existing immunity, a phenomenon known as antigenic drift [1]. As a result, vaccine formulations for the prevention of influenza infection and reduction of severe disease must be constantly updated to identify candidate vaccine viruses that stimulate antibodies able to neutralise the range of antigenically diverse viruses circulating in any given season. Other factors may also influence influenza vaccine effectiveness (VE), including age, immunocompetence and history of influenza infection [2, 3, 4, 5, 6], while immunogenicity and effectiveness may be reduced among persons who have been repeatedly vaccinated [7, 8]. Annual estimates of influenza VE therefore provide important post‐marketing evaluation of the degree to which seasonal influenza vaccines prevent medically attended influenza disease, provided laboratory viral confirmation is undertaken. This information can in turn be considered when updating vaccine formulations [9, 10] and for public health purposes. In addition, VE estimates can inform estimates of influenza burden averted as a result of influenza vaccination [11, 12, 13].

The aim of this study was to examine influenza VE in Australia across three seasons spanning 2017–2019, with variable timing of the epidemic period and distribution of influenza subtypes and lineages. This approach allowed us to explore seasonal differences and the impact of age, influenza type and influenza vaccination history on influenza VE prior to the COVID‐19 pandemic.

## 2 Material and Methods

### 2.1 Study Design

The study used a test‐negative design to estimate influenza VE [14, 15]. Data were included from two sentinel surveillance networks that monitored influenza‐like illness in general practice in Australia during 2017–2019—the Australian Sentinel Practices Research Network (ASPREN) and the Victorian Sentinel Practice Influenza Network (VicSPIN) [16, 17]. The VicSPIN network was restricted to surveillance in the state of Victoria, while ASPREN performed surveillance Australia‐wide with low sampling of Victoria. ASPREN operated year‐round, while VicSPIN collected data only during seasonal periods (weeks 18–45 in 2017, weeks 18–43 in 2018 and weeks 18–44 in 2019). Similarities in the methods used by the two surveillance networks enabled pooling of data and the estimation of influenza VE nationwide [18]. Previous estimates of VE have been published from these networks, including interim estimates for 2017 and 2019 [18, 19, 20].

The ASPREN and VicSPIN data were pooled for each year of the study period (2017, 2018 and 2019). Patients presenting to a participating general practitioner (GP), or for ASPREN, a nurse practitioner (NP), with influenza‐like illness (ILI: fever, cough, fatigue) were asked to provide nasal and throat swabs (ideally nasopharyngeal). During 2017, ASPREN GPs used a systematic sampling approach, collecting specimens from the first three patients presenting with ILI each week during weeks 18 to 44, and the first patient presenting with ILI during other weeks. The same approach was used during 2018 and 2019, with additional enhanced surveillance including all adults aged 65 years and older with ILI, where practical. No sampling limitations were used by VicSPIN GPs, who were encouraged to test all ILI patients where possible. Demographic data and information about each patient's medical history and vaccination status were obtained by GPs/NPs from patient medical records or self‐report. For ASPREN GPs using the FluSync tool, patient vaccination records were cross‐checked by ASPREN staff to verify or supplement the recorded status. Mandatory reporting of influenza vaccinations to the Australian Immunisation Register (AIR) was not introduced until 2021 and thus was not a source of vaccination status at the time of this study.

### 2.2 Vaccination

In Australia, influenza vaccination is recommended for everyone, and under the National Immunisation Program (NIP) is provided free‐of‐charge to some groups (Table S1). During the study period, these groups included adults aged 65 years and older, pregnant women, Aboriginal and Torres Strait Islander people aged at least 15 years and people with certain high‐risk medical conditions (Table S1). Eligibility for free vaccination for Aboriginal and Torres Strait Islander children was expanded to include those aged 6 months to < 5 years in 2018 and those aged 5–14 years in 2019. In addition, many states provided free vaccination to all children aged < 5 years in 2018 and 2019, and all children in this age group were added to the NIP in 2020 [21].

All standard‐dose vaccines used in 2017 and for individuals aged < 65 years in 2018–2019 were quadrivalent inactivated formulations. In 2018, high‐dose and adjuvanted trivalent inactivated vaccines were introduced for older adults aged 65 years and older, and in 2019, only adjuvanted trivalent vaccines were provided for this age group. The vaccine viruses for each year are shown in Table 1.

**TABLE 1:** Influenza vaccine reference viruses and associated clades used for influenza vaccines in Australia during 2016–2019.

Influenza type/subtype	2016	2017	2018^a^	2019^b^
A(H1N1)pdm09	A/California/7/2009	A/Michigan/45/2015 (clade 6B.1)	A/Michigan/45/2015 (clade 6B.1)	A/Michigan/45/2015 (clade 6B.1)
A(H3N2)	A/Hong Kong/4801/2014 (clade 3C.2a)	A/Hong Kong/4801/2014 (clade 3C.2a)	A/Singapore/INFIMH‐16‐0019/2016 (clade 3C.2a1)	A/Switzerland/8060/2017 (clade 3C.2a2)
B/Victoria	B/Brisbane/60/2008 (clade V1A)	B/Brisbane/60/2008 (clade V1A)	B/Brisbane/60/2008 (clade V1A)	B/Colorado/06/2017^c^ (clade V1A.1)
B/Yamagata	B/Phuket/3073/2013 (clade Y3)	B/Phuket/3073/2013 (clade Y3)	B/Phuket/3073/2013^c^ (clade Y3)	B/Phuket/3073/2013 (clade Y3)

^a^ Trivalent high‐dose or adjuvanted vaccine used for adults 65 years and older.

^b^ Trivalent adjuvanted vaccine used for adults 65 years and older.

^c^ Influenza B lineage included in trivalent formulations used for older adults in 2018 and 2019.

### 2.3 Specimen Collection and Laboratory Methods

Respiratory swabs were collected by GPs and NPs. For ASPREN, practitioners used MWE Sigma Virocult (MW951PF2ML) flocked swabs (Medical Wire and Equipment, United Kingdom) in 2 mL of viral transport medium (VTM), while VicSPIN GPs used Copan flocked swabs (Copan Diagnostics, USA) in 3 mL of universal transport medium (UTM). Samples were sent for testing at SA Pathology for samples collected by ASPREN practitioners or the Victorian Infectious Diseases Reference Laboratory (VIDRL) for samples collected by VicSPIN GPs. Both laboratories used in‐house multiplex real‐time RT‐PCR assays for the detection of influenza type and subtype, as well as other respiratory pathogens (Table S2).

Influenza‐positive specimens from both laboratories were forwarded to the WHO Collaborating Centre for Reference and Research on Influenza in Melbourne for further characterisation. Influenza virus isolation was attempted on received samples using Madin‐Darby canine kidney (MDCK) or MDCK‐SIAT cells (A(H3N2) viruses only). Antigenic similarity of virus isolates to their relevant vaccine antigen (Table 1) was assessed in haemagglutination inhibition (HI) assays using post‐infection ferret antisera, as previously described [22, 23, 24, 25]. Isolates were defined as antigenically similar to the vaccine virus if the antisera titre required to inhibit virus haemagglutination was within fourfold of the homologous vaccine virus‐antisera titre. To identify the genomic diversity of viruses, genetic sequencing was performed using Sanger or Next Generation Sequencing (NGS), as described previously [23, 24, 25]. Phylogenetic analysis was performed using the maximum likelihood method and generalised‐time reversible (GTR) model as implemented in IQ‐TREE2 version 2.3.3 [26]. Trees were visualised using ggtree version 3.10.1 [27].

### 2.4 Eligibility Criteria

Based on the test‐negative design, cases were identified as patients who tested positive for influenza using real‐time RT‐PCR, and non‐cases were patients who tested negative for influenza virus. Individuals were considered vaccinated if they had received the vaccine at least 14 days prior to symptom onset. Influenza vaccines are not licensed for use in infants aged < 6 months, so children < 1 year were excluded. Patients were also excluded if their vaccination status was unknown, there were fewer than 14 days from vaccination to the date of symptom onset or there were more than 8 days from symptom onset to the date of swab collection. If the date of vaccination or symptom onset was unknown, it was assumed that GPs had followed protocol, and therefore, patients were still eligible to be included in the analysis.

### 2.5 Study Period

VE was measured for the period of epidemic activity in each season, defined as commencing when a positive case had been identified for two consecutive weeks at least 2 weeks after the seasonal influenza vaccine became available under the NIP, and ending after the epidemic peak when there had been no influenza cases reported for at least 2 weeks, or at the estimated date of vaccine expiry (assigned as mid‐February), whichever was earlier. The timing of vaccine availability under the NIP was based on publicly available information, and for the three seasons of the study period assigned as 13 April 2017, 18 April 2018 and 17 April 2019 respectively [28, 29, 30]. Based on this, epidemic periods were defined as weeks 16 to 48 for 2017, weeks 18 2018 to 07 2019 for 2018 and weeks 16 to 52 for 2019. In 2018–2019, high inter‐seasonal influenza activity had been reported from several jurisdictions [31, 32].

### 2.6 Statistical Analyses

Patient characteristics were tabulated for each year with stratification by influenza type/subtype and vaccination status. Chi‐square tests were used to investigate differences between groups within each year.

VE was estimated by comparing the odds of the exposure (vaccination) among individuals testing positive and negative for influenza using logistic regression. The odds ratio (OR) was converted to a VE estimate using the formula VE = 1—OR_adj_ × 100%. Estimates were adjusted for age (modelled as age plus age‐squared) and week of sample collection (modelled as a natural cubic spline with three knots). VE was calculated overall and stratified by age, for each influenza type and subtype or lineage, and with clade‐specific estimates where possible. We also estimated VE based on previous vaccination status for each influenza type and subtype/lineage. VE estimates were not reported if the number of expected vaccinated cases or non‐cases from the Chi‐squared approximation was fewer than four. All statistical analyses were undertaken in R (version 4.2.0).

### 2.7 Sensitivity Analysis

Data collection for the VicSPIN network was restricted to the usual seasonal periods expected for that state (see ‘Study design’). To examine the effect of this difference between the networks, a sensitivity analysis was undertaken with restriction to cases and non‐cases swabbed during the VicSPIN data collection period. VE was estimated as described above.

### 2.8 Ethics

ASPREN data were collected and de‐identified in accordance with the National Health Security Act 2007. Furthermore, consent forms were introduced for ASPREN from 2018, in accordance with the requirements of The Royal Australian College of General Practitioners (RACGP) National Research and Evaluation Ethics Committee (NREEC 18‐003). Collection, use, and reporting of VicSPIN data were undertaken in accordance with the Victorian Public Health and Wellbeing Act 2008 and the Public Health and Wellbeing Regulations 2009. Patients were provided with an English‐language information sheet, and consented verbally to the study. As a result, overarching human research ethics committee approval was not required for this study.

## 3 Results

There were 3302, 2256 and 3895 patients who consented to the collection of swabs in 2017, 2018 and 2019 respectively. Of these, 423 (13%) were excluded from the analysis for 2017, 283 (13%) for 2018 and 524 (13%) for 2019 due to age < 1 year, unknown vaccination status, < 14 days from vaccination to symptom onset or > 8 days from symptom onset to swab collection (Table S3).

**FIGURE 1:**
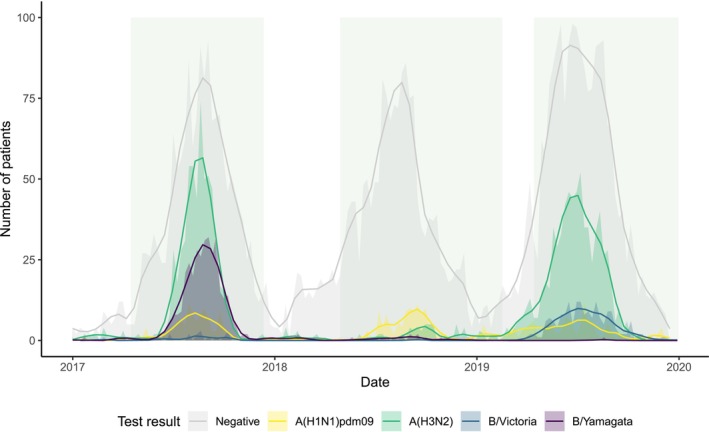
Weekly influenza cases and negative tests identified by ASPREN and VicSPIN in Australia during 2017–2019. Viruses with unknown subtype/lineage and mixed influenza types are not shown. Shaded rectangles indicate the periods of epidemic influenza activity used to estimate vaccine effectiveness in each of the influenza seasons. For each epidemic, the number of patients is shown for each virus in the shaded area, while the lines show the smoothed average using geom_smooth.

Influenza activity during the study period is depicted in Figure 1. The 2017 and 2019 seasons were characterised by higher percentages of positive influenza tests (43% and 34% respectively) than in 2018 (12%) (Table S4). During 2017, A(H3N2) viruses predominated (50% of confirmed influenza patients), followed by B/Yamagata‐lineage viruses (29%). Influenza A viruses were predominant during 2018, with 59% identified as A(H1N1)pdm09 and 27% identified as A(H3N2). A(H3N2) viruses were again predominant during 2019 (64%), and there was co‐circulation of B/Victoria‐lineage (17%) and A(H1N1)pdm09 (11%) viruses.

Patient characteristics by influenza status are detailed in Table 2. Among included patients, the proportion vaccinated across the 3 years of the study comprised 32% in 2017, 42% in 2018 and 40% in 2019 (Table 2). In all 3 years, a greater proportion of women than men were vaccinated, and people aged 65 years and over had the highest vaccinated proportion, with more than 70% vaccinated (Table S4). Among other age groups, vaccine coverage was lowest in 2017, although the proportion vaccinated increased substantially for 2018 and 2019, especially among those aged 0–17 years (6% in 2017, 21% in 2018 and 2019). VE for any influenza type and across all age groups varied depending on the predominant virus and was 37% (95% CI 22, 48) in 2017, 53% (95% CI 33, 67) in 2018 and 50% (95% CI 40, 58) in 2019 (Figure S1).

**TABLE 2:** Patient characteristics by influenza status for Australia during periods of epidemic influenza activity in 2017–2019.

		2017			2018			2019		
		Negative	Positive	*p*	Negative	Positive	*p*	Negative	Positive	*p*
Total		1555	1158	—	1579	224	—	2006	1022	—
Sex	Female	876 (56%)	619 (53%)	0.14	924 (59%)	118 (53%)	0.10	1142 (57%)	509 (50%)	< 0.01
	Male	679 (44%)	539 (47%)		655 (41%)	106 (47%)		864 (43%)	513 (50%)	
Age group	0–17 years	274 (18%)	305 (26%)	< 0.01	295 (19%)	54 (24%)	< 0.01	337 (17%)	326 (32%)	< 0.01
	18–64 years	1056 (68%)	706 (61%)		1000 (63%)	152 (68%)		1314 (66%)	544 (53%)	
	65+ years	225 (14%)	147 (13%)		284 (18%)	18 (8.0%)		355 (18%)	152 (15%)	
State^a^	Australian Capital Territory	30 (1.9%)	24 (2.1%)	< 0.01	15 (0.9%)	0 (0%)	< 0.01	42 (2.1%)	22 (2.2%)	< 0.01
	New South Wales	468 (30%)	363 (31%)		420 (27%)	53 (24%)		555 (28%)	303 (30%)	
	Northern Territory	26 (1.7%)	23 (2.0%)		6 (0.4%)	0 (0%)		18 (0.9%)	18 (1.8%)	
	Queensland	176 (11%)	164 (14%)		253 (16%)	32 (14%)		284 (14%)	149 (15%)	
	South Australia	219 (14%)	140 (12%)		287 (18%)	32 (14%)		266 (13%)	92 (9.0%)	
	Tasmania	60 (3.9%)	63 (5.4%)		89 (5.6%)	4 (1.8%)		119 (5.9%)	33 (3.2%)	
	Victoria	377 (24%)	300 (26%)		261 (17%)	44 (20%)		413 (21%)	225 (22%)	
	Western Australia	198 (13%)	81 (7.0%)		248 (16%)	59 (26%)		309 (15%)	180 (18%)	
Vaccination status	Unvaccinated	1004 (65%)	852 (74%)	< 0.01	883 (56%)	166 (74%)	< 0.01	1085 (54%)	720 (70%)	< 0.01
	Vaccinated	551 (35%)	306 (26%)		696 (44%)	58 (26%)		921 (46%)	302 (30%)	
High risk group^b^	Yes	457 (29%)	288 (25%)	0.01	483 (31%)	55 (25%)	0.06	611 (30%)	255 (25%)	< 0.01
	No/Unknown	1098 (71%)	870 (75%)		1096 (69%)	169 (75%)		1395 (70%)	767 (75%)	

^a^ Unknown: *n* = 1 for 2017.

^b^ Includes medical comorbidities, obesity, pregnancy, and people who identify as Aboriginal or Torres Strait Islander.

### 3.1 A(H1N1)pdm09

**FIGURE 2:**
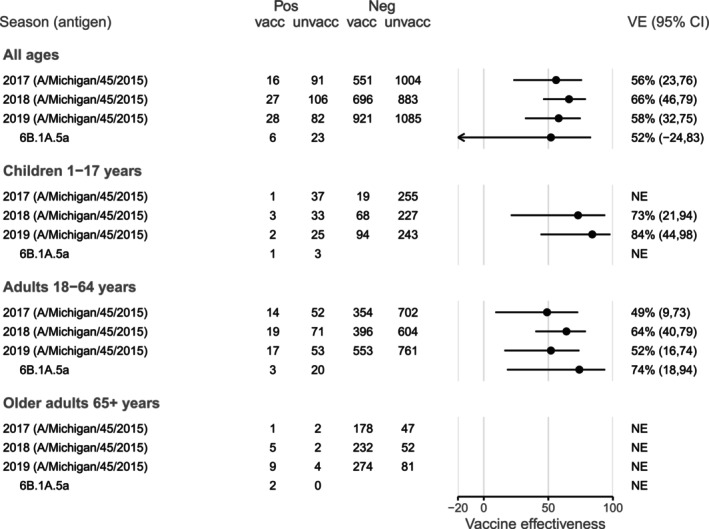
Vaccine effectiveness for influenza A(H1N1)pdm09 in Australia during 2017–2019. People aged 65 years and older received high‐dose or adjuvanted vaccine in 2018 and adjuvanted vaccine in 2019. NE: not estimated if Chi‐square approximation estimated an expected count < 4 in any cell of the 2 × 2 table of vaccination by case status.

In all years, a majority of A(H1N1)pdm09 viruses were antigenically similar in HI assay to both the cell‐ and egg‐propagated A/Michigan/45/2015 reference antigens (Table S5). During 2017, all A(H1N1)pdm09 viruses with sequencing results available were in clades 6B.1 and 6B.1A, with the vaccine virus, A/Michigan/45/2015, in the 6B.1 clade. Genetic diversity increased in subsequent years, with predominance of the 6B.1A.5a clade in 2019 (Table S6; Figure S2). VE estimates were similar for A(H1N1)pdm09 across all three seasons: 56% (95% CI 23, 76) for 2017, 66% (95% CI 46, 79) for 2018 and 58% (95% CI 32, 75) for 2019 (Figure 2), and remained stable when estimation was restricted to the VicSPIN data collection period, weeks 18–44 (Table S7). Where estimation by age group was possible, VE point estimates were higher among children than among adults aged 18–64 years; however, there were too few cases to estimate VE for older adults in any year. HA phylogenetic clade‐specific estimates were possible in 2019 for the 6B.1A.5a clade, with VE estimated at 52% (95% CI −24, 83] (Figure 2; Table S8), reflecting clade similarity. In 2017 and 2018, VE estimates were lower among people who had received vaccination in both the current and prior years compared with the current year only, although with substantial overlap of confidence intervals (52% [95% CI 12, 75] and 84% [95% CI 23, 99] for 2017, 61% [95% CI 36, 77] and 90% [95% CI 69, 98] for 2018); while in 2019, VE was similar for these groups (66% [95% CI 41, 81] versus 68% [95% CI 21, 91]) (Figure 5).

### 3.2 A(H3N2)

Influenza A(H3N2) predominated in 2017 and 2019. Antigenic similarity to the vaccine virus varied across the years, with only 51% of 2017 viruses well‐inhibited by the cell‐propagated reference post‐infection ferret antisera and 66% by the egg‐propagated reference post‐infection ferret antisera (Table S5). In 2019, 63% of A(H3N2) viruses were well‐inhibited by the cell‐propagated reference antisera, but all viruses were low reactors against the egg‐propagated reference virus (Table S5). Genetic sequencing data indicated greater diversity of A(H3N2) viruses in 2017, consistent with lower antigenic similarity against the cell‐propagated reference antisera in that year. There was a reduction in the number of HA phylogenetic clades detected in later years (Table S6; Figure S3).

**FIGURE 3:**
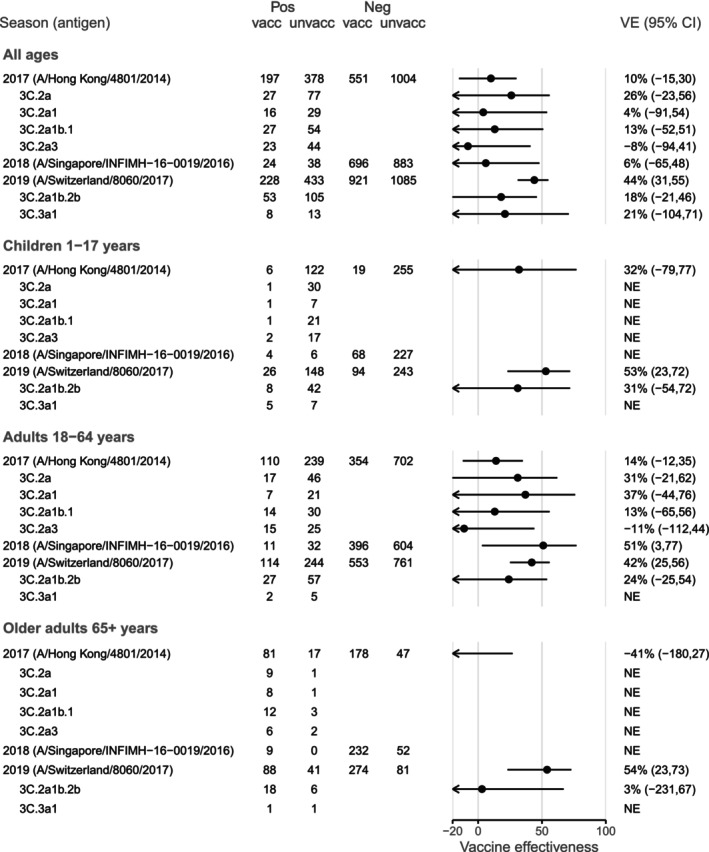
Vaccine effectiveness for influenza A(H3N2) in Australia during 2017–2019. People aged 65 years and older received high‐dose or adjuvanted vaccine in 2018 and adjuvanted vaccine in 2019. NE: not estimated if Chi‐square approximation estimated an expected count < 4 in any cell of the 2 × 2 table of vaccination by case status.

VE point estimates for A(H3N2) were low during 2017 (10% [95% CI −15, 30]) and 2018 (6% [95% CI −65, 48]), but comparatively higher in 2019 (44% [95% CI 31, 55]) (Figure 3). These remained similar when estimation was restricted to weeks 18–44 (Table S7). As with A(H1N1)pdm09, point estimates for 2017 were highest for children (32% [95% CI −79, 77]). In 2017, point estimates for older adults were very low (−41% [95% CI −180, 27]), but in 2019, when adjuvanted vaccine was used in this age group, the VE was somewhat higher than for adults aged 18–64 years (54% [95% CI 23, 73] compared with 42% [95% CI 25, 56]), and similar to the estimate for children (53% [95% CI 23, 72]). HA clade‐specific VE estimates for 2017 were higher for clade 3C.2a (the vaccine virus clade), compared with clades 3C.2a1, 3C.2a1b.1 and 3C.2a3 (Figure 3; Table S8). During 2019, point estimates for all ages were similar for clades 3C.2a1b.2b and 3C.3a1, despite the vaccine being in the 3C.2a2 clade (Figure 3; Table S8).

Results for VE by prior year's vaccination status are presented in Figure 5. In all years, VE point estimates were higher among people vaccinated in the current year only compared with those vaccinated only in the prior year or in both years. This trend was apparent regardless of whether the vaccine virus changed. However, in 2017, when the vaccine virus did not change, the point estimate for people vaccinated in both years was lower than for those vaccinated in the previous year only; while in 2018 and 2019, when the vaccine virus was updated, the point estimates for the ‘Prior’ and ‘Both’ groups were similar (Figure 5).

### 3.3 Influenza B

**FIGURE 4:**
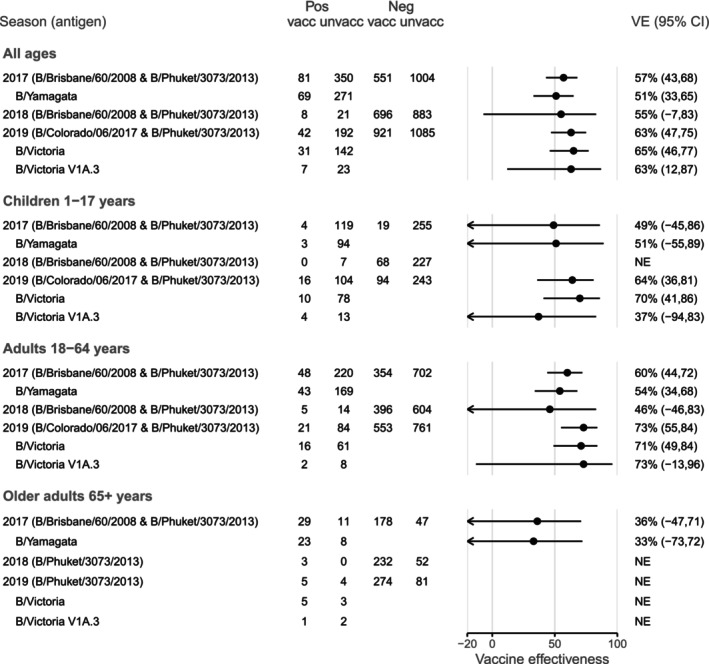
Vaccine effectiveness for influenza B in Australia during 2017–2019. People aged 65 years and older received high‐dose or adjuvanted trivalent vaccine in 2018 and adjuvanted trivalent vaccine in 2019. NE: not estimated if Chi‐square approximation estimated an expected count < 4 in any cell of the 2 × 2 table of vaccination by case status.

Antigenic similarity to the vaccine virus was variable across the study period. Most B/Yamagata‐lineage viruses in 2017 were antigenically similar to both the cell‐ and egg‐propagated B/Phuket/3073/2013 reference antigens (Table S5). For B/Victoria‐lineage viruses in 2019, 83% and 89% of viruses were well inhibited by the cell‐ and egg‐propagated reference post‐infection ferret antisera respectively (Table S5). There was limited diversity among both influenza B lineages, with the majority of B/Victoria‐lineage viruses being part of the V1A.3 HA phylogenetic clade, and all the B/Yamagata‐lineage viruses falling into the Y3 HA phylogenetic clade (Table S6; Figures S4 and S5). VE was relatively consistent for influenza B, estimated at 57% (95% CI 43, 68) in 2017, 55% (95% CI ‐7, 83) in 2018 and 63% (95% CI 47, 75) in 2019, although confidence intervals were very wide in 2018 with reduced circulation of influenza B (Figure 4). This was despite changes in the predominant lineage, with more B/Yamagata‐lineage viruses in 2017, few influenza B detections in 2018 and a predominance of B/Victoria‐lineage viruses in 2019. Estimation for adults aged 18–64 years was possible in all 3 years and point estimates ranged from 46% to 73% (Figure 4). Estimates for children were not possible in 2018, while in 2017 and 2019, the point estimates for children were somewhat lower than for adults aged 18–64 years (Figure 4). Lineage‐specific point estimates for B/Yamagata in 2017 and B/Victoria in 2019 were similar to the overall influenza B estimates (Figure 4; Table S9), consistent with the dominance of each of these lineages in those seasons. Clade‐specific estimation was possible for B/Victoria in 2019 and indicated similar VE for clade V1A.3 than for B/Victoria overall (63% [95% CI 12, 87] compared with 65% [95% CI 46, 77]) (Figure 4; Table S9). Of note, any older adults contributing to this estimate would have been vaccinated with a B/Yamagata‐lineage‐containing vaccine.

**FIGURE 5:**
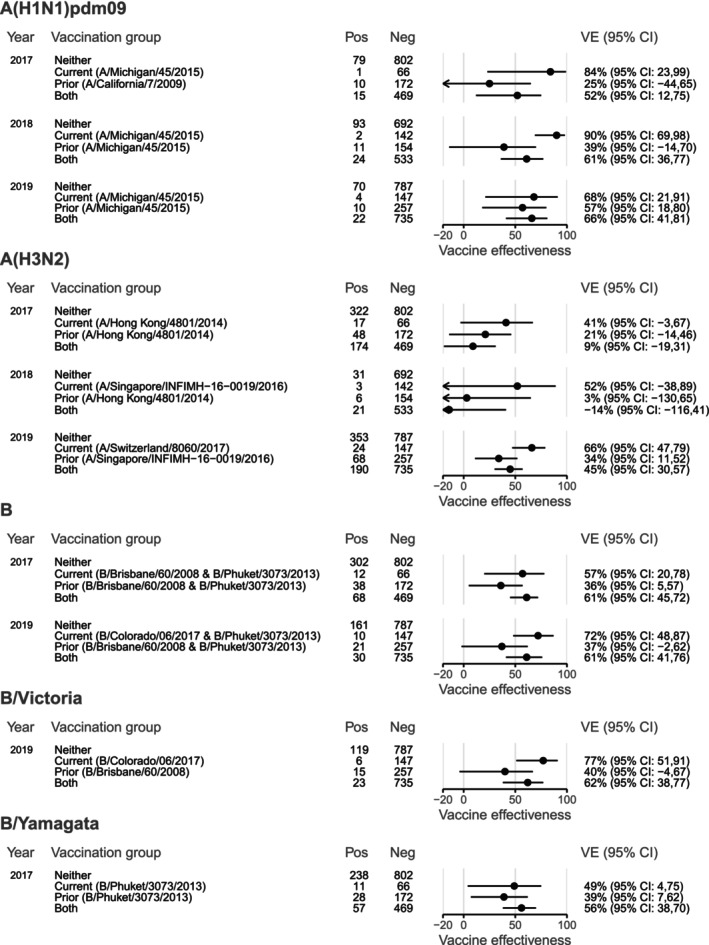
Influenza vaccine effectiveness by prior vaccination status in Australia during 2017–2019. Note that for the 2019 estimates for influenza B and B/Victoria, people aged 65 years and older would have received a B/Yamagata‐containing trivalent inactivated vaccine in both 2018 and 2019.

Considering prior vaccination status, VE point estimates for the 2017 season were highest against influenza B overall and against B/Yamagata‐lineage viruses for the group vaccinated in both 2016 and 2017, during which the vaccine antigen did not change (Table 1; Figure 5). However, in 2019, the group vaccinated in the current year only, who had received the vaccine containing the new B/Colorado/06/2017‐like antigen and had the highest VE point estimates for both influenza B overall and B/Victoria‐lineage viruses. Nevertheless, in both years, VE was lowest for the group that did not get vaccinated in the current year (‘Prior’) (Figure 5).

## 4 Discussion

This study used data pooled across two Australian general practice surveillance networks to estimate seasonal influenza VE by subtype, age and vaccination history during the 2017–2019 influenza seasons. These seasons differed markedly in terms of case numbers and predominant virus type/subtype. The 2017 season saw the highest peak in influenza activity, predominated by influenza A(H3N2) with co‐circulation of B/Yamagata‐lineage viruses. In 2018, there was low influenza activity, with A(H1N1)pdm09 being predominant. The 2019 season was again predominated by A(H3N2), with co‐circulation of B/Victoria‐lineage and, to a lesser extent, A(H1N1)pdm09 viruses. The age distribution of influenza patients was similar across the 3 years, although there were slightly higher proportions of children and older adults with influenza identified in 2019. Influenza VE varied and was generally higher for A(H1N1)pdm09 and influenza B viruses, but lower for A(H3N2).

For influenza A(H1N1)pdm09, most circulating viruses were in the same HA phylogenetic clade as the vaccine antigen during 2017, but not during 2018 or 2019. However, antigenic data did not indicate lower reactivity among circulating A(H1N1)pdm09 viruses compared with the egg‐grown vaccine virus in 2018 or 2019. It is worth noting that post‐infection ferret antisera raised to A(H1N1)pdm09 viruses have in the past not detected reduced reactivity in some age groups, whereas reduced reactivity has been detected in post‐vaccination human sera, possibly due to their influenza priming history [33]. Nevertheless, VE estimates were comparable across the years, ranging from 49% to 64% for adults aged 18–64 years, albeit with wide confidence intervals (Figure 2). Compared with estimates for the northern hemisphere 2017–2018 season, during which the same vaccine antigen was used, our 2018 estimate of 64% (95% CI 40, 79) for adults aged 18–64 years was higher than the VE reported in the United States (48% [95% CI 18, 67] for 18–49 years and 36% [95% CI ‐23, 67] for 50–64 years [34]) and Europe (50% [95% CI 28, 66] for 15–64 years) [35]. The vaccine antigen for A(H1N1)pdm09 used in Australia in 2019 was the same as that used in the northern hemisphere during the 2018–2019 season. Among adults aged 18–64 years, our VE estimate of 52% (95% CI 16, 74) was slightly higher than estimates reported from the United States (43% [95% CI 28, 55] for 18–49 years and 30% [95% CI 6, 48] for 50–64 years [36]), but comparable with Europe (51% [95% CI ‐1, 76] for hospitalisations and 50% [95% CI 9, 74] for primary care visits, both among people aged 18–64 years [37]), but lower than interim estimates from Canada (68% [95% CI 51, 80] for 20–64 years) [38].

In general, VE was lower for A(H3N2) than for the other vaccine antigens. More frequent antigenic drift of A(H3N2) viruses results in greater diversity of circulating viruses [39], making it difficult to identify a candidate vaccine virus in advance that is capable of providing broad coverage across all circulating genetic variants. VE for A(H3N2) was lowest in 2017 and 2018 at 10% (95% CI −15, 30) and 6% (95% CI −65, 48) respectively (Figure 3). Genetic HA sequencing data indicated that over one‐quarter of A(H3N2) viruses in 2017 were in the 3C.2a group (28% of viruses with a clade available), with the remainder distributed across seven different HA clades. Antigenic data indicated about half of the viruses were not well inhibited by the cell‐grown vaccine post‐infection ferret antisera. Furthermore, low VE in 2017 could have been exacerbated by the absence of a putative glycosylation site at position 160 of the HA protein in the egg‐propagated A/Hong Kong/4801/2014 vaccine virus. This glycosylation site was present in most circulating A(H3N2) viruses and inhibits effective antibody binding [40, 41]. Finally, in 2017, repeat vaccination effects may have hindered overall VE, as that was the second year in which individuals received the A/Hong Kong/4801/2014‐containing vaccine [6, 7]. It has been hypothesised that sequential vaccination reduces VE when there is no change in vaccine composition, but there is increasing antigenic distance between the vaccine and circulating viruses [8, 42]. In our study, this was most evident for older adults for whom VE was very low, in which the majority were repeatedly vaccinated. Low VE was also reported from the 2017–2018 northern hemisphere season, when A/Hong Kong/4801/2014 was included in the vaccine for a second year, with VE of 14% (95% CI −8, 31) in Canada [43], 13% (95% CI −21, 38] in Europe [35] and 22% (95% CI 12, 31) in the United States [34]. This suggests that even when other data do not suggest a change in the vaccine virus is needed, the risk of a lower VE might be sufficient impetus to update the vaccine.

Although the A(H3N2) vaccine virus was updated in 2018 to A/Singapore/INFIMH‐16‐0019/2016 (clade 3C.2a1), that virus continued to harbour problematic mutations, which may have contributed to low VE. In addition, there was mismatch between the HA clade of the vaccine virus (3C.2a1) and the majority of circulating viruses, which were mainly distributed in clades 3C.2a1b.1, 3C.2a1b.2 and 3C.2a2 (22/29, 76% of viruses with genetic analysis) (Table S6). We observed too few cases to reliably estimate VE by HA clade that year. However, estimation was possible among adults aged 18–64 years (51% [95% CI 3, 77]; Figure 3) and was higher compared with adult estimates from the 2018–2019 northern hemisphere season, during which the same vaccine virus (A/Singapore/INFIMH‐16‐0019/2016) was used. For example, VE for the 2018–2019 season in the United States was 3% (95% CI 0, 24) among adults aged 18–49 years, and 0% (95% CI 0, 18) among adults aged 50–64 years [36]. Similarly in Europe, VE for the 2018–2019 season was −26% (95% CI −66, 4) among adults aged 15–64 years [44].

In contrast to 2017 and 2018, VE for A(H3N2) in 2019 improved, and notably, estimates for older adults were not worse than for adults aged 18–64 years. In that year, adults aged 65 years and over were eligible for the adjuvanted vaccine, a change to the Australian NIP that was introduced after the 2017 epidemic, which saw very low VE and a number of deaths in this age group [45]. VE for older adults is often low [46], which may be due to a decreased ability to generate an immune response to vaccination, repeated vaccination effects, and increased susceptibility to severe influenza disease [47, 48]. Thus, the use of the adjuvanted vaccine in 2019 appears to have improved VE. It was not possible to directly compare our 2019 VE estimates for A(H3N2) to those obtained in the northern hemisphere, as the A/Switzerland/8060/2017 virus was only used in southern hemisphere vaccines.

Lower VE for influenza A(H3N2) is an ongoing concern, exacerbated by factors such as more rapid antigenic drift compared with other influenza viruses [49], and more frequently occurring egg adaptations that alter antigenicity [41], combined with the attenuating effects of repeated influenza vaccination [7, 50, 51]. Strategies are needed to address these issues, which can include the use of high‐dose and adjuvanted vaccines, and alternative vaccine production methods such as recombinant HA vaccines [52, 53, 54].

For influenza B, there was no change in the B/Yamagata‐like vaccine virus used across the study period. However, B/Brisbane/60/2008 (clade V1A) was used for the B/Victoria‐like virus in 2017 and 2018, with a change to B/Colorado/06/2017 (clade V1A.1) for the 2019 formulation. Despite this, and despite a change in predominant lineage from Victoria to Yamagata between 2017 and 2019, VE against influenza B was consistent across all 3 years of the study (range 55%–63%) (Figure 4). VE estimates for influenza B were generally comparable to other regions across the study period, although somewhat higher than for some 2017–2018 European (range 36–54%) and 2018–2019 US (34%, 95% CI 0, 62) estimates [34, 36, 55, 56, 57]. Clade‐specific estimates for B/Victoria clade V1A.3 in 2019 were slightly lower than the overall influenza B estimate (63% [95% CI 12, 87] versus 63% [95% CI 47, 75]) (Figure 4; Table S8). Viruses in this clade, which predominated in 2019, harboured a triple deletion in their HA, which was not present in the vaccine virus, and were poorly inhibited by post‐infection ferret antisera raised to the egg‐propagated V1A.1 vaccine virus. There was less evidence of an effect of prior vaccination among people infected with influenza B viruses, which may be due to cross‐lineage protection. However, inconsistent use of trivalent and quadrivalent vaccines poses challenges in estimating the effects of vaccination against B‐lineage viruses [7, 58].

There are several important public health considerations in understanding and interpreting VE and vaccination timing. Circulating influenza viruses evolve constantly, necessitating seasonal updates to the vaccine's formulation. The antigen composition is reviewed annually for each hemisphere, with the influenza vaccine composition decision‐making process including review of interim (mid‐season) and final (prior season) VE estimates. As a result, ongoing monitoring of influenza VE is essential in providing information about how current vaccines are performing, which may in turn provide insight into the performance of proposed candidate vaccine viruses for a coming season. Following vaccination, the duration of protection is an important consideration, and there is evidence of waning protection over time post‐vaccination, especially among older populations [59, 60]. Thus, there are important policy considerations associated with aligning influenza vaccination with the start of the influenza season, particularly for groups at higher risk of morbidity and mortality [61]. That said, because the exact timing of influenza seasons remains unpredictable, vaccination campaigns tend to start around the same time each year (around April in Australia), and vaccination can sometimes occur too early or too late to maximise protection [62].

This study has some limitations. Data were pooled across two surveillance networks. Although the clinical case definitions and data collection approaches were aligned, differences in data collection and recording may persist. There may have been errors in the recording of vaccination status, which was largely obtained by self‐report. Moreover, some participants were not able to be included in the analysis due to missing data for vaccination status, which particularly limited estimates based on prior vaccination status. We assumed that GPs had followed protocol and therefore included participants if their date of vaccination, symptom onset or swab collection was unknown, which may have biased results if our assumption was incorrect. Some subgroup analyses were affected by small sample sizes, resulting in wide confidence intervals and limited power for VE estimation. Expanded surveillance, recruiting a larger number of patients, would mitigate this problem.

## 5 Conclusions

Influenza activity across the 2017–2019 seasons in Australia was markedly different and associated with variable influenza VE. Although influenza vaccination provides benefits compared with no vaccination, there is a need for ongoing research and surveillance to improve the ability to select vaccine viruses with optimal effectiveness. Results from this study demonstrate the ongoing value of influenza surveillance systems in Australia to enable the estimation of influenza VE over time and across different population groups by influenza type/subtype, lineage and phylogenetic HA clade.

## Author Contributions

**Tanya Diefenbach‐Elstob:** conceptualization, methodology, formal analysis, data curation, writing – review and editing, writing – original draft. **Monique B. Chilver:** investigation, resources, data curation, writing – review and editing, project administration. **Violeta Spirkoska:** investigation, writing – review and editing, resources, data curation. **Kylie S. Carville:** investigation, data curation, resources, project administration, writing – review and editing. **Clyde Dapat:** investigation, data curation, writing – review and editing, visualization. **Mark Turra:** investigation, resources, writing – review and editing. **Thomas Tran:** investigation, resources, writing – review and editing. **Yi‐Mo Deng:** investigation, writing – review and editing, resources. **Heidi Peck:** investigation, writing – review and editing, resources. **Ian G. Barr:** conceptualization, resources, writing – review and editing, supervision. **Nigel Stocks:** conceptualization, writing – review and editing, resources, supervision. **Sheena G. Sullivan:** supervision, conceptualization, methodology, formal analysis, data curation, writing – review and editing, project administration.

## Ethics Statement

ASPREN data were collected and de‐identified in accordance with the National Health Security Act 2007. Furthermore, consent forms were introduced for ASPREN from 2018, in accordance with the requirements of The Royal Australian College of General Practitioners (RACGP) National Research and Evaluation Ethics Committee (NREEC 18–003). Collection, use, and reporting of VicSPIN data were undertaken in accordance with the Victorian Public Health and Wellbeing Act 2008 and the Public Health and Wellbeing Regulations 2009. Patients were provided with an English‐language information sheet and consented verbally to the study. As a result, overarching human research ethics committee approval was not required for this study.

## Consent

ASPREN data were collected and de‐identified in accordance with the National Health Security Act 2007. Furthermore, consent forms were introduced for ASPREN from 2018, in accordance with the requirements of The Royal Australian College of General Practitioners (RACGP) National Research and Evaluation Ethics Committee (NREEC 18‐0003). Collection, use, and reporting of VicSPIN data were undertaken in accordance with the Victorian Public Health and Wellbeing Act 2008 and the Public Health and Wellbeing Regulations 2009. Patients were provided with an English‐language information sheet and consented verbally to the study.

## Conflicts of Interest

IGB holds shares in a vaccine producing company; SGS reports paid consulting and advisory board participation for vaccine manufacturers, including Astra‐Zeneca, CSL Seqirus, Sanofi, GSK, Moderna, Novavax and Pfizer. The WHO Collaborating Centre for Reference and Research on Influenza receives funding from the International Federation of Pharmaceutical Manufacturers and CSL Seqirus for the development of influenza vaccines.

## Supporting information


**Table S1:** Eligibility for free influenza vaccination in Australia, 2017–2019.
Table S2: Testing for respiratory pathogens by SA Pathology and VIDRL during 2017–2019.
Table S3: Participant exclusions.
Table S4: Patient characteristics by vaccination status for Australia during periods of influenza epidemic activity for 2017–2019.
Table S5: Antigenic analysis using the haemagglutination inhibition (HI) assay for isolates of viruses collected by ASPREN and VicSPIN in Australia during 2017–2019.
Table S6: HA phylogenetic clade analysis of viruses collected by ASPREN and VicSPIN in Australia during 2017–2019. Percentages are calculated based on grouping by year and vaccination status.
Table S7: Sensitivity analysis of vaccine effectiveness estimates by year and age for influenza in Australia during 2017–2019. Vaccine effectiveness estimates are not shown for clade and year groups with insufficient cases and non‐cases to attempt an estimate. The sensitivity analysis was restricted to cases and non‐cases swabbed during the VicSPIN data collection period.
Table S8: HA phylogenetic clade‐specific vaccine effectiveness estimates by year and age for influenza A(H1N1pdm09) and A(H3N2). Vaccine effectiveness estimates are not shown for clade and year groups with insufficient cases and non‐cases to attempt an estimate for at least the ‘All ages’ group.
Table S9: Lineage‐ and HA phylogenetic clade‐specific vaccine effectiveness estimates by year and age for influenza B. Vaccine effectiveness estimates are not shown for HA clade and year groups with insufficient cases and non‐cases to attempt an estimate for at least the ‘All ages’ group.
Figure S1: Overall vaccine effectiveness estimates against any influenza in Australia for 2017–2019 by age group and year. NE: not estimated if Chi‐square approximation estimated an expected count < 4 in any cell of the 2 × 2 table of vaccination by case status.
Figure S2: Phylogenetic tree for A(H1N1)pdm09 viruses collected by ASPREN and VicSPIN in Australia during 2017–2019. Viruses are coloured by collection date (taxa legend), with details of their associated clade. State, age group, vaccination status and antigenic characteristics are coloured according to the heatmap legend.
Figure S3: Phylogenetic tree for A(H3N2) viruses collected by ASPREN and VicSPIN in Australia during 2017–2019. Viruses are coloured by collection date (taxa legend), with details of their associated clade. State, age group, vaccination status and antigenic characteristics are coloured according to the heatmap legend.
Figure S4: Phylogenetic tree for B/Victoria‐lineage viruses collected by ASPREN and VicSPIN in Australia during 2017–2019. Viruses are coloured by collection date (taxa legend), with details of their associated clade. State, age group, vaccination status and antigenic characteristics are coloured according to the heatmap legend.
Figure S5: Phylogenetic tree for B/Yamagata‐lineage viruses collected by ASPREN and VicSPIN in Australia during 2017–2019. Viruses are coloured by collection date (taxa legend), with details of their associated clade. State, age group, vaccination status and antigenic characteristics are coloured according to the heatmap legend.

## Data Availability

Data may be made available upon reasonable request to the corresponding author.
