# Supplementary material for: Influenza Vaccine Effectiveness in Australia During 2017–2019
Source: Influenza Other Respir Viruses. 2025 Jul 16;19(7):e70137. doi: 10.1111/irv.70137 (PMC12266806; doi:10.1111/irv.70137)
Supplement: Supplementary file 1 — Table S1: Eligibility for free influenza vaccination in Australia, 2017–2019. Table S2: Testing for respiratory pathogens by SA Pathology and VIDRL during 2017–2019. Table S3: Participant exclusions. Table S4: Patient characteristics by vaccination status for Australia during periods of influenza epidemic activity for 2017–2019. Table S5: Antigenic analysis using the haemagglutination inhibition (HI) assay for isolates of viruses collected by ASPREN and VicSPIN in Australia during 2017–2019. Table S6: HA phylogenetic clade analysis of viruses collected by ASPREN and VicSPIN in Australia during 2017–2019. Percentages are calculated based on grouping by year and vaccination status. Table S7: Sensitivity analysis of vaccine effectiveness estimates by year and age for influenza in Australia during 2017–2019. Vaccine effectiveness estimates are not shown for clade and year groups with insufficient cases and non‐cases to attempt an estimate. The sensitivity analysis was restricted to cases and non‐cases swabbed during the VicSPIN data collection period. Table S8: HA phylogenetic clade‐specific vaccine effectiveness estimates by year and age for influenza A(H1N1pdm09) and A(H3N2). Vaccine effectiveness estimates are not shown for clade and year groups with insufficient cases and non‐cases to attempt an estimate for at least the ‘All ages’ group. Table S9: Lineage‐ and HA phylogenetic clade‐specific vaccine effectiveness estimates by year and age for influenza B. Vaccine effectiveness estimates are not shown for HA clade and year groups with insufficient cases and non‐cases to attempt an estimate for at least the ‘All ages’ group. Figure S1: Overall vaccine effectiveness estimates against any influenza in Australia for 2017–2019 by age group and year. NE: not estimated if Chi‐square approximation estimated an expected count < 4 in any cell of the 2 × 2 table of vaccination by case status. Figure S2: Phylogenetic tree for A(H1N1)pdm09 viruses collected by ASPREN and VicSPIN [file IRV-19-e70137-s001.pdf]

Supplementary Table 1: Eligibility for free influenza vaccination in Australia, 2017-2019.

Year	National Immunisation Program (NIP)	State programs
2017	• Aboriginal and/or Torres Strait Islander people aged 6 months to <5 years and ≥15 years<br>• Adults aged 65 years and older<br>• People aged ≥6 months with certain medical conditions which increase the risk of influenza disease complications<br>• Pregnant women	• WA: Children aged 6 months to <5 years
2018	• 2017 groups +<br>• Adults aged ≥65 years and older can receive high dose or adjuvanted trivalent vaccines	• ACT, NSW, QLD, SA, TAS, VIC, WA: Children aged 6 months to <5 years
2019	• 2018 groups +<br>• Aboriginal and/or Torres Strait Islander children aged 5-14 years<br>• All children aged 6 months to <5 years<br>• Adults aged ≥65 years can receive high dose or adjuvanted trivalent vaccines	• ACT, NSW, NT, QLD, VIC, SA, TAS, WA: Children aged 6 months to <5 years

References

Department of Health. Australian Technical Advisory Group on Immunisation (ATAGI) advice for immunisation providers regarding the administration of seasonal influenza vaccines in 2017. Available from: https://www.nitag-resource.org/sites/default/files/072023d24d6488e9aa1b65f1a1d59b812646d907_1.pdf

Department of Health. Statement on the administration of seasonal influenza vaccines in 2018. Available from: https://www.health.gov.au/resources/publications/atagi-advice-on-seasonal-influenza-vaccines-in-2018

Department of Health. Statement on the administration of seasonal influenza vaccines in 2019. Available from: https://www.health.gov.au/resources/publications/atagi-advice-on-seasonal-influenza-vaccines-in-2019

Howard ZL, Dalton CB, Carlson S, Baldwin Z, Durrheim DN. Impact of funding on influenza vaccine uptake in Australian children. *Public Health Res Pract*. 2021;31(1). doi: 10.17061/phrp3112104.

National Centre for Immunisation Research & Surveillance. Significant events in influenza vaccination practice in Australia. Available from: https://ncirs.org.au/sites/default/files/2020-07/Influenza-history-July%202020_0.pdf

Queensland Health. Immunisation Program Update 2018 Influenza Special Edition. Issue No.11. April 2018. Available from: https://www.health.qld.gov.au/__data/assets/pdf_file/0029/704891/qhip-news-18iss11.pdf.

SA Health. Frequently Asked Questions: Immunisation for Health Care Workers in South Australia Policy Directive 2017. Available from: https://www.sahealth.sa.gov.au/wps/wcm/connect/c5d3b28042de6261b83cfe8cd21c605e/FINAL+-+HCW+Immunisation+FAQs++v1.1+January+2018.pdf?MOD=AJPERES&CACHEID=ROOTWORKSPACE-c5d3b28042de6261b83cfe8cd21c605e-nKOAYw5.

Victoria State Government Department of Health. 2017 influenza outbreak. Available from: https://www.health.vic.gov.au/your-health-report-of-the-chief-health-officer-victoria-2018/communicable-disease/2017-influenza.

Supplementary Table 2: Testing for respiratory pathogens by SA Pathology and VIDRL during 2017-2019.

**Pathogen**	**SA Pathology**	**VIDRL**
Influenza A	Yes	Yes
Influenza B	Yes	Yes
Influenza C	No	Yes
Respiratory syncytial virus	Yes	Yes
Parainfluenza 1	Yes	Yes
Parainfluenza 2	Yes	Yes
Parainfluenza 3	Yes	Yes
Adenovirus	Yes	Yes
Enterovirus	No	Yes
Rhinovirus	Yes	Yes
Human metapneumovirus	Yes	Yes
Mycoplasma pneumoniae	Yes	No
Bordetella pertussis	Yes	No
Seasonal coronaviruses (non-SARS-CoV-2)	No	Yes

SA: South Australia; VIDRL: Victorian Infectious Diseases Reference Laboratory

Supplementary Table 3: Participant exclusions

Reason	2017		2018		2019	
	**ASPREN**	**VicSPIN**	**ASPREN**	**VicSPIN**	**ASPREN**	**VicSPIN**
All patients	2632	670	1955	301	3192	703
Age <1 year	16 (0.6%)	0 (0%)	24 (1.2%)	1 (0.3%)	34 (1.1%)	2 (0.3%)
Unknown vaccination status	25 (0.9%)	64 (9.6%)	17 (0.9%)	6 (2.0%)	38 (1.2%)	14 (2.0%)
<14 days from vaccination to symptom onset	20 (0.8%)	15 (2.2%)	49 (2.5%)	15 (5.0%)	135 (4.2%)	45 (6.4%)
>8 days from symptom onset to swab	258 (9.8%)	37 (5.5%)	163 (8.3%)	27 (9.0%)	227 (7.1%)	61 (8.7%)
**Patients included***	**2323 (88%)**	**556 (83%)**	**1715 (88%)**	**258 (86%)**	**2779 (87%)**	**592 (84%)**
Outside epidemic period	166 (6.3%)	-	170 (8.7%)	-	343 (10.7%)	-
Patients included in VE estimates	2157 (82%)	556 (83%)	1545 (79%)	258 (86%)	2436 (76%)	592 (84%)

*participants may be excluded for more than one reason

Supplementary Table 4: Patient characteristics by vaccination status for Australia during periods of influenza epidemic activity for 2017-2019.

		2017			2018			2019		
		**UV**	**V**	**p**	**UV**	**V**	**p**	**UV**	**V**	**p**
**Total**		1856	857	-	1049	754	-	1805	1223	-
**Sex**	Female	986 (53%)	509 (59%)	<0.01	564 (54%)	478 (63%)	<0.01	903 (50%)	748 (61%)	<0.01
	Male	870 (47%)	348 (41%)		485 (46%)	276 (37%)		902 (50%)	475 (39%)	
**Age group**	0-17 years	546 (29%)	33 (3.9%)	<0.01	274 (26%)	75 (9.9%)	<0.01	525 (29%)	138 (11%)	<0.01
	18-64 years	1233 (66%)	529 (62%)		721 (69%)	431 (57%)		1150 (64%)	708 (58%)	
	65+ years	77 (4.1%)	295 (34%)		54 (5.1%)	248 (33%)		130 (7.2%)	377 (31%)	
**State***	Australian Capital Territory	40 (2.2%)	14 (1.6%)	<0.01	7 (0.7%)	8 (1.1%)	<0.01	39 (2.2%)	25 (2.0%)	<0.01
	New South Wales	608 (33%)	223 (26%)		281 (27%)	192 (25%)		525 (29%)	333 (27%)	
	Northern Territory	36 (1.9%)	13 (1.5%)		4 (0.4%)	2 (0.3%)		20 (1.1%)	16 (1.3%)	
	Queensland	236 (13%)	104 (12%)		149 (14%)	136 (18%)		245 (14%)	188 (15%)	
	South Australia	244 (13%)	115 (13%)		177 (17%)	142 (19%)		208 (12%)	150 (12%)	
	Tasmania	60 (3.2%)	63 (7.4%)		42 (4.0%)	51 (6.8%)		83 (4.6%)	69 (5.6%)	
	Victoria	450 (24%)	227 (27%)		188 (18%)	117 (16%)		351 (19%)	287 (23%)	
	Western Australia	182 (9.8%)	97 (11%)		201 (19%)	106 (14%)		334 (19%)	155 (13%)	
**Influenza status**	Negative	1004 (54%)	551 (64%)	<0.01	883 (84%)	696 (92%)	<0.01	1085 (60%)	921 (75%)	<0.01
	A(H1N1)pdm09	91 (4.9%)	16 (1.9%)		106 (10%)	26 (3.4%)		82 (4.5%)	28 (2.3%)	
	A(H3N2)	378 (20%)	197 (23%)		38 (3.6%)	23 (3.1%)		430 (24%)	228 (19%)	
	A(Mixed)	-	-		-	-		3 (0.2%)	0 (0%)	
	A(NS)	33 (1.8%)	12 (1.4%)		1 (<0.1%)	1 (0.1%)		10 (0.6%)	4 (0.3%)	
	B/Victoria	16 (0.9%)	0 (0%)		1 (<0.1%)	0 (0%)		142 (7.9%)	31 (2.5%)	
	B/Yamagata	271 (15%)	69 (8.1%)		13 (1.2%)	4 (0.5%)		1 (<0.1%)	0 (0%)	
	B/NL	63 (3.4%)	12 (1.4%)		7 (0.7%)	3 (0.4%)		49 (2.7%)	11 (0.9%)	
	A/B(Mixed)	-	-		-	-		3 (0.2%)	0 (0%)	
	C	-	-		0 (0%)	1 (0.1%)		-	-	
**Previous vaccination status**	Neither	1531 (82%)	-	-	835 (80%)	-	-	1381 (77%)	-	-
	Prior	275 (15%)	-		174 (17%)	-		359 (20%)	-	
	Current	-	99 (12%)		-	148 (20%)		-	186 (15%)	
	Both	-	734 (86%)		-	584 (77%)		-	980 (80%)	
	Unknown	50 (2.7%)	24 (2.8%)		40 (3.8%)	22 (2.9%)		65 (3.6%)	57 (4.7%)	

*unknown: n=1 for 2017; NL: no lineage; NS: no subtype; UV: unvaccinated; V: vaccinated

Supplementary Table 5: Antigenic analysis using the haemagglutination inhibition (HI) assay for isolates of viruses collected by ASPREN and VicSPIN in Australia during 2017-2019.

**Virus**	**Year**	**Vaccine virus**	**Cell-propagated**		**Egg-propagated**	
			**Antigenically similar**	**Low reacting***	**Antigenically similar**	**Low reacting***
**A(H1N1)pdm09**	2017	A/Michigan/45/2015	58 (100%)	0 (0%)	58 (100%)	0 (0%)
	2018	A/Michigan/45/2015	90 (100%)	0 (0%)	90 (100%)	0 (0%)
	2019	A/Michigan/45/2015	61 (100%)	0 (0%)	59 (97%)	2 (3%)
**A(H3N2)**	2017	A/Hong Kong/4801/2014	23 (51%)	22 (49%)	62 (66%)	32 (34%)
	2018	A/Singapore/INFIMH-16-0019/2016	27 (96%)	1 (4%)	1 (33%)	2 (67%)
	2019	A/Switzerland/8060/2017	112 (63%)	66 (37%)	0 (0%)	221 (100%)
**B/Victoria**	2017	B/Brisbane/60/2008	6 (86%)	1 (14%)	0 (0%)	7 (100%)
	2018	B/Colorado/06/2017[†]	0 (0%)	0 (0%)	0 (0%)	0 (0%)
	2019	B/Colorado/06/2017[†]	39 (83%)	8 (17%)	5 (11%)	42 (89%)
**B/Yamagata**	2017	B/Phuket/3073/2013	111 (100%)	0 (0%)	108 (97%)	3 (3%)
	2018	B/Phuket/3073/2013	9 (100%)	0 (0%)	2 (22%)	7 (78%)
	2019	B/Phuket/3073/2013	1 (100%)	0 (0%)	0 (0%)	1 (100%)

*Low reacting means the HI titre for the tested virus was more than 4-fold lower than the homologous titre, suggesting poor inhibition of the virus by ferret antisera; [†]not included in adjuvanted or high dose trivalent inactivated vaccine among elderly

Supplementary Table 6: HA phylogenetic clade analysis of viruses collected by ASPREN and VicSPIN in Australia during 2017-2019. Percentages are calculated based on grouping by year and vaccination status.

Clade	2017		2018		2019	
	UV (n=1856)	V (n=857)	UV (n=1049)	V (n=754)	UV (n=1805)	V (n=1223)
**A(H1N1)pdm09**						
6B.1	13 (0.7)	1 (0.1)	-	-	-	-
6B.1A	4 (0.2)	-	7 (0.7)	1 (0.1)	-	-
6B.1A.1	-	-	5 (0.5)	4 (0.5)	2 (0.1)	-
6B.1A.2	-	-	6 (0.6)	1 (0.1)	1 (0.1)	-
6B.1A.5	-	-	4 (0.4)	1 (0.1)	-	-
6B.1A.5a	-	-	3 (0.3)	-	23 (1.3)	6 (0.5)
6B.1A.5a.1	-	-	-	-	5 (0.3)	2 (0.2)
6B.1A.5b	-	-	2 (0.2)	-	6 (0.3)	1 (0.1)
6B.1A.6	-	-	2 (0.2)	1 (0.1)	-	-
**Total**	**17**	**1**	**29**	**8**	**37**	**9**
**A(H3N2)**						
3C.2a	77 (4.1)	27 (3.2)	-	-	-	-
3C.2a1	29 (1.6)	16 (1.9)	1 (0.1)	2 (0.3)	-	-
3C.2a1b	9 (0.5)	9 (1.1)	-	-	-	-
3C.2a1b.1	54 (2.9)	27 (3.2)	5 (0.5)	2 (0.3)	-	-
3C.2a1b.1b	-	-	-	-	4 (0.2)	2 (0.2)
3C.2a1b.2	-	-	4 (0.4)	4 (0.5)	8 (0.4)	2 (0.2)
3C.2a1b.2a	-	-	-	-	1 (0.1)	1 (0.1)
3C.2a1b.2b	-	-	1 (0.1)	-	105 (5.8)	53 (4.3)
3C.2a2	26 (1.4)	26 (3.0)	5 (0.5)	2 (0.3)	-	-
3C.2a3	44 (2.4)	23 (2.7)	-	-	-	-
3c.3a	1 (0.1)	-	-	-	-	-
3C.3a1	-	-	2 (0.2)	1 (0.1)	13 (0.7)	8 (0.7)
**Total**	**240**	**128**	**18**	**11**	**131**	**66**
**B/Victoria**						
V1A	1 (0.1)	-	-	-	2 (0.1)	-
V1A.1	1 (0.1)	-	-	-	2 (0.1)	1 (0.1)
V1A.2	-	-	-	-	2 (0.1)	-
V1A.3	-	-	-	-	23 (1.3)	7 (0.6)
**Total**	**2**				**29**	**8**
**B/Yamagata**						
Y3	6 (0.3)	5 (0.6)	4 (0.4)	1 (0.1)	1 (0.1)	-

UV: unvaccinated; V: vaccinated

Supplementary Table 7: Sensitivity analysis of vaccine effectiveness estimates by year and age for influenza in Australia during 2017-2019. Vaccine effectiveness estimates are not shown for clade and year groups with insufficient cases and non-cases to attempt an estimate. The sensitivity analysis was restricted to cases and non-cases swabbed during the VicSPIN data collection period.

**Type and subtype**	**Age group**	**Year**	**Cases**		**Non-cases**		**VE % (95% CI)**
			**UV**	**V**	**UV**	**V**	
**A or B**	All ages	2017	849	305	950	524	37 (23, 48)
		2018	133	48	752	599	54 (33, 69)
		2019	685	297	991	887	50 (40, 59)
	0-17 years	2017	290	14	246	18	35 (-41, 70)
		2018	43	6	192	59	56 (-6, 84)
		2019	272	43	231	90	60 (40, 74)
	18-64 years	2017	529	175	661	336	40 (25, 52)
		2018	88	27	513	337	60 (38, 75)
		2019	374	152	692	532	51 (39, 61)
	65+ years	2017	30	116	43	170	-7 (-89, 39)
		2018	2	15	47	203	-
		2019	39	102	68	265	52 (21, 71)
**A(H1N1)pdm09**	All ages	2017	91	16	950	524	56 (23, 76)
		2018	90	24	752	599	66 (44, 80)
		2019	69	24	991	887	63 (38, 78)
	0-17 years	2017	37	1	246	18	-
		2018	30	3	192	59	69 (3, 93)
		2019	22	1	231	90	91 (53, 99)
	18-64 years	2017	52	14	661	336	49 (9, 73)
		2018	58	16	513	337	64 (38, 81)
		2019	46	14	692	532	60 (27, 79)
	65+ years	2017	2	1	43	170	-
		2018	2	5	47	203	-
		2019	1	9	68	265	-
**A(H3N2)**	All ages	2017	377	197	950	524	10 (-15, 29)
		2018	24	17	752	599	8 (-83, 55)
		2019	418	228	991	887	44 (30, 55)
	0-17 year	2017	122	6	246	18	32 (-79, 77)
		2018	5	3	192	59	-
		2019	144	26	231	90	52 (22, 71)
	18-64 years	2017	238	110	661	336	14 (-13, 34)
		2018	19	6	513	337	56 (-9, 84)
		2019	239	114	692	532	42 (25, 55)
	65+ years	2017	17	81	43	170	-41 (-181, 27)
		2018	0	8	47	203	-
		2019	35	88	68	265	54 (22, 73)
**B**	All ages	2017	348	80	950	524	58 (43, 69)
		2018	18	8	752	599	45 (-37, 80)
		2019	186	41	991	887	63 (47, 75)
	0-17 year	2017	118	4	246	18	48 (-49, 86)
		2018	7	0	192	59	-
		2019	102	16	231	90	64 (36, 81)
	18-64 years	2017	219	48	661	336	60 (44, 72)

		2018	11	5	513	337	34 (-90, 80)
		2019	81	21	692	532	72 (55, 84)
	65+ years	2017	11	28	43	170	39 (-41, 72)
		2018	0	3	47	203	-
		2019	3	4	68	265	80 (-42, 97)
**B/Victoria**	All ages	2019	137	30	991	887	64 (46, 77)
	0-17 year	2019	76	10	231	90	71 (42, 86)
	18-64 years	2019	59	16	692	532	70 (49, 84)
	65+ years	2019	2	4	68	265	-
**B/Yamagata**	All ages	2017	269	68	950	524	52 (34, 65)
	0-17 year	2017	93	3	246	18	50 (-60, 89)
	18-64 years	2017	168	43	661	336	53 (33, 68)
	65+ years	2017	8	22	43	170	36 (-66, 73)

Supplementary Table 8: HA phylogenetic clade-specific vaccine effectiveness estimates by year and age for influenza A(H1N1pdm09) and A(H3N2). Vaccine effectiveness estimates are not shown for clade and year groups with insufficient cases and non-cases to attempt an estimate for at least the 'All ages' group.

Type and subtype	Clade	Year	Age group	Cases		Non-cases		VE % (95% CI)
				UV	V	UV	V	
**A(H1N1)pdm09**	**6B.1A.5a**	**2019**	All ages	23	6	1085	921	52 (-24, 83)
			0-17 years	3	1	243	94	-
			18-64 years	20	3	761	553	74 (18, 94)
			65+ years	0	2	81	274	-
**A(H3N2)**	**3C.2a**	**2017**	All ages	77	27	1004	551	26 (-23, 56)
			0-17 years	30	1	255	19	-
			18-64 years	46	17	702	354	31 (-21, 62)
			65+ years	1	9	47	178	-
	**3C.2a1**		All ages	29	16	1004	551	4 (-91, 54)
			0-17 years	7	1	255	19	-
			18-64 years	21	7	702	354	37 (-44, 76)
			65+ years	1	8	47	178	-
	**3C.2a1b.1**		All ages	54	27	1004	551	13 (-52, 51)
			0-17 years	21	1	255	19	-
			18-64 years	30	14	702	354	13 (-65, 56)
			65+ years	3	12	47	178	-
	**3C.2a3**		All ages	44	23	1004	551	-8 (-94, 41)
			0-17 years	17	2	255	19	-
			18-64 years	25	15	702	354	-11 (-112, 44)
			65+ years	2	6	47	178	-
	**3C.2a1b.2b**	**2019**	All ages	105	53	1085	921	18 (-21, 46)
			0-17 years	42	8	243	94	31 (-54, 72)
			18-64 years	57	27	761	553	24 (-25, 54)
			65+ years	6	18	274	81	3 (-231, 67)
	**3C.3a1**		All ages	13	8	1085	921	21 (-104, 71)
			0-17 years	7	5	243	94	-
			18-64 years	5	2	761	553	-
			65+ years	1	1	81	274	-

Supplementary Table 9: Lineage- and HA phylogenetic clade-specific vaccine effectiveness estimates by year and age for influenza B. Vaccine effectiveness estimates are not shown for HA clade and year groups with insufficient cases and non-cases to attempt an estimate for at least the ‘All ages’ group.

**Type and lineage**	**Clade**	**Year**	**Age group**	**Cases**		**Non-cases**		**VE % (95% CI)**
				**UV**	**V**	**UV**	**V**	
**B/Victoria**	**Any**	**2019**	All ages	142	31	1085	921	65 (46, 77)
			0-17 years	78	10	243	94	70 (41, 86)
			18-64 years	61	16	761	553	71 (49, 84)
			65+ years	3	5	81	274	-
	**V1A.3**	**2019**	All ages	23	7	1085	921	63 (12, 87)
			0-17 years	13	4	243	94	37 (-94, 83)
			18-64 years	8	2	761	553	73 (-13, 96)
			65+ years	2	1	81	274	-
**B/Yamagata**	**Any**	**2017**	All ages	271	69	1004	551	51 (33, 65)
			0-17 years	94	3	255	19	51 (-55, 89)
			18-64 years	169	43	702	354	54 (34, 68)
			65+ years	8	23	47	178	33 (-73, 72)

Season (antigen)	Pos vacc	Pos unvacc	Neg vacc	Neg unvacc	VE (95% CI)
**All ages**					
2017	306	852	551	1004	37% (22,48)
2018	58	166	696	883	53% (33,67)
2019	302	720	921	1085	50% (40,58)
**Children 1-17 years**					
2017	14	291	19	255	35% (-39,70)
2018	7	47	68	227	56% (3,83)
2019	44	282	94	243	60% (40,74)
**Adults 18-64 years**					
2017	175	531	354	702	41% (26,53)
2018	35	117	396	604	59% (39,73)
2019	155	389	553	761	50% (38,60)
**Older adults 65+ years**					
2017	117	30	178	47	-7% (-88,39)
2018	16	2	232	52	NE
2019	103	49	274	81	57% (29,74)

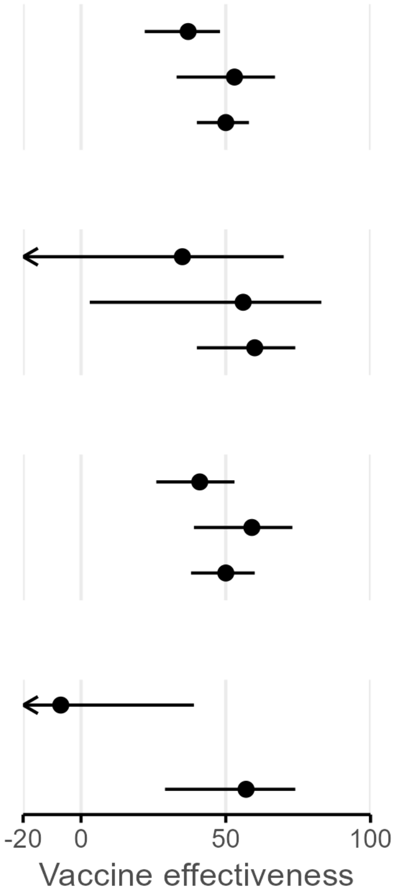

Supplementary Figure 1: Overall vaccine effectiveness estimates against any influenza in Australia for 2017-2019 by age group and year. NE: not estimated if Chi-square approximation estimated an expected count <4 in any cell of the 2x2 table of vaccination by case status.

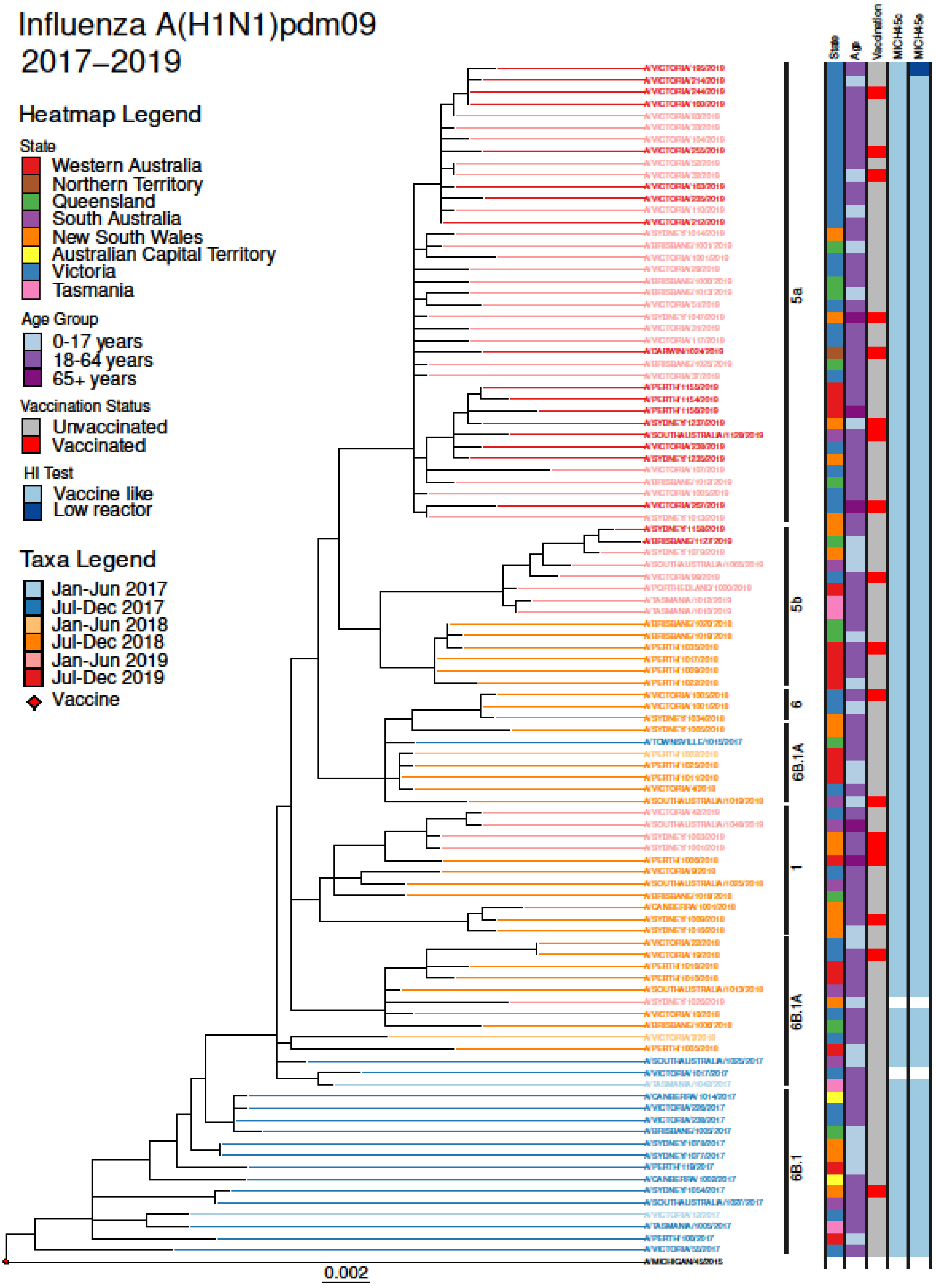

Supplementary Figure 2: Phylogenetic tree for A(H1N1)pdm09 viruses collected by ASPREN and VicSPIN in Australia during 2017-2019. Viruses are coloured by collection date (taxa legend), with details of their associated clade. State, age group, vaccination status, and antigenic characteristics are coloured according to the heatmap legend.

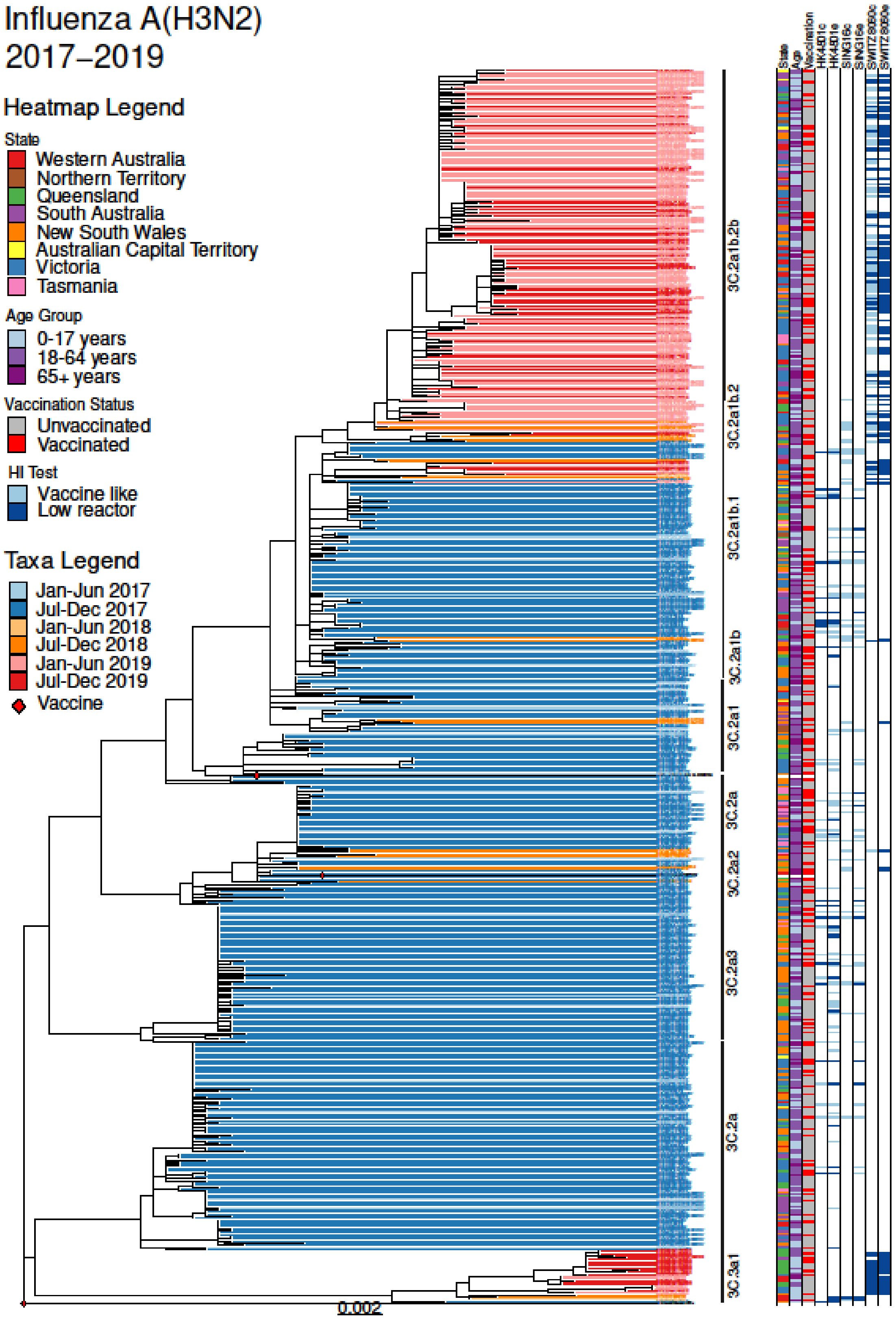

Supplementary Figure 3: Phylogenetic tree for A(H3N2) viruses collected by ASPREN and VicSPIN in Australia during 2017-2019. Viruses are coloured by collection date (taxa legend), with details of their associated clade. State, age group, vaccination status, and antigenic characteristics are coloured according to the heatmap legend.

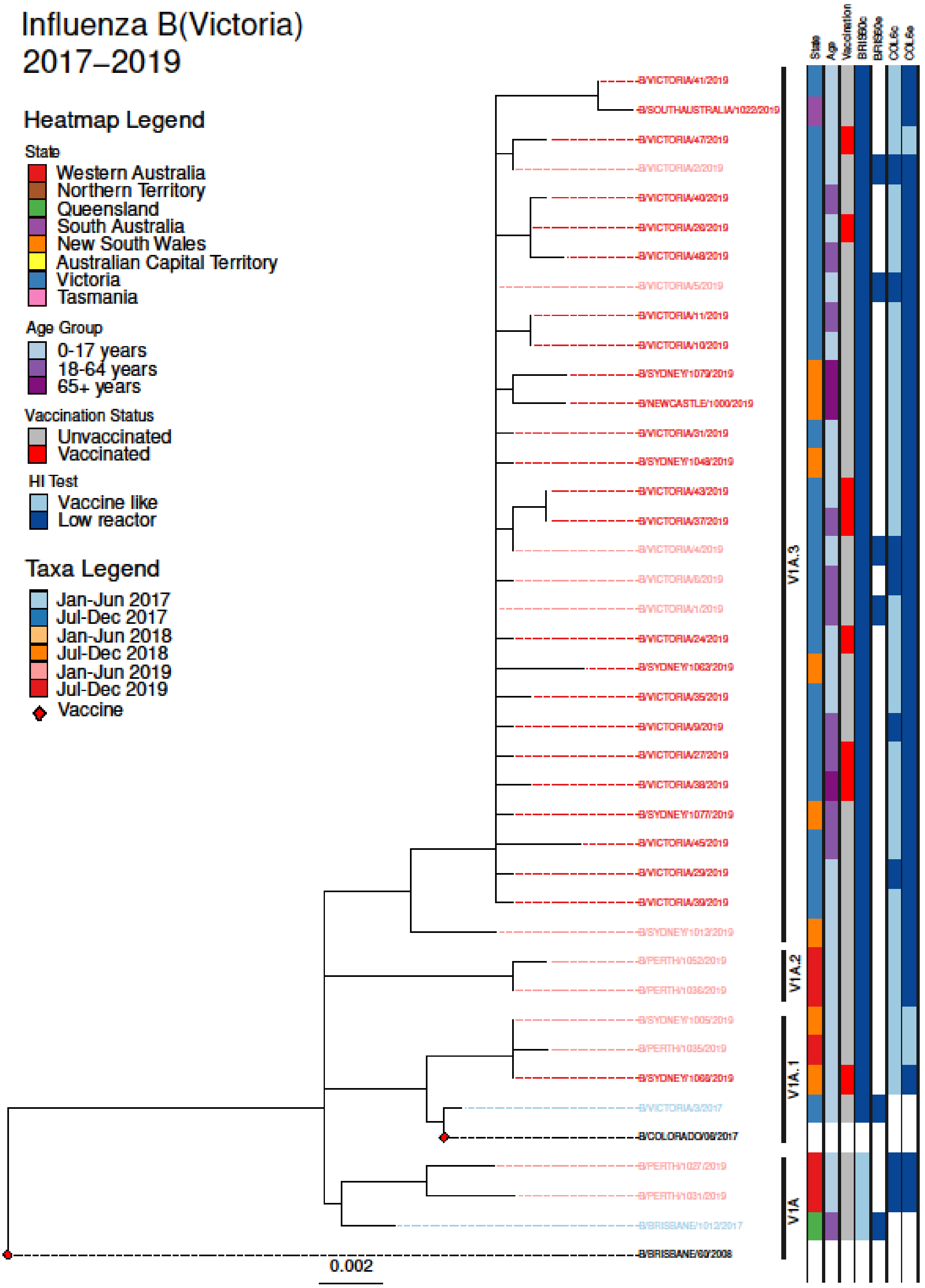

Supplementary Figure 4: Phylogenetic tree for B/Victoria-lineage viruses collected by ASPREN and VicSPIN in Australia during 2017-2019. Viruses are coloured by collection date (taxa legend), with details of their associated clade. State, age group, vaccination status, and antigenic characteristics are coloured according to the heatmap legend.

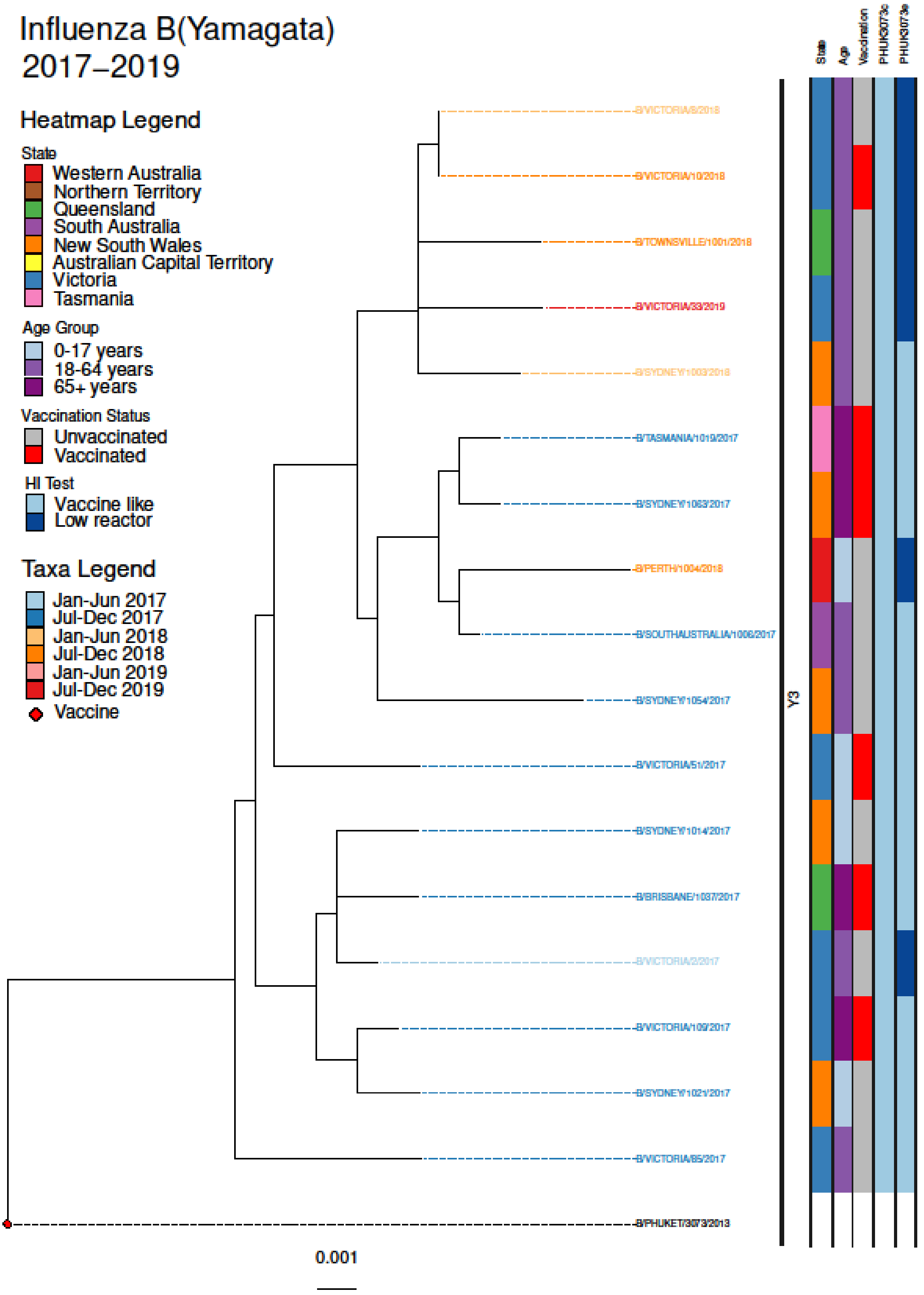

Supplementary Figure 5: Phylogenetic tree for B/Yamagata-lineage viruses collected by ASPREN and VicSPIN in Australia during 2017-2019. Viruses are coloured by collection date (taxa legend), with details of their associated clade. State, age group, vaccination status, and antigenic characteristics are coloured according to the heatmap legend.
